# Supplementary material for: An Enhanced Method for the Use of Reptile Skin Sheds as a High-Quality DNA Source for Genome Sequencing
Source: Genes (Basel). 2023 Aug 25;14(9):1678. doi: 10.3390/genes14091678 (PMC10531129; doi:10.3390/genes14091678)
Supplement: Supplementary file 1 [file genes-14-01678-s001.zip › Supplementary_Figure_final.pdf]

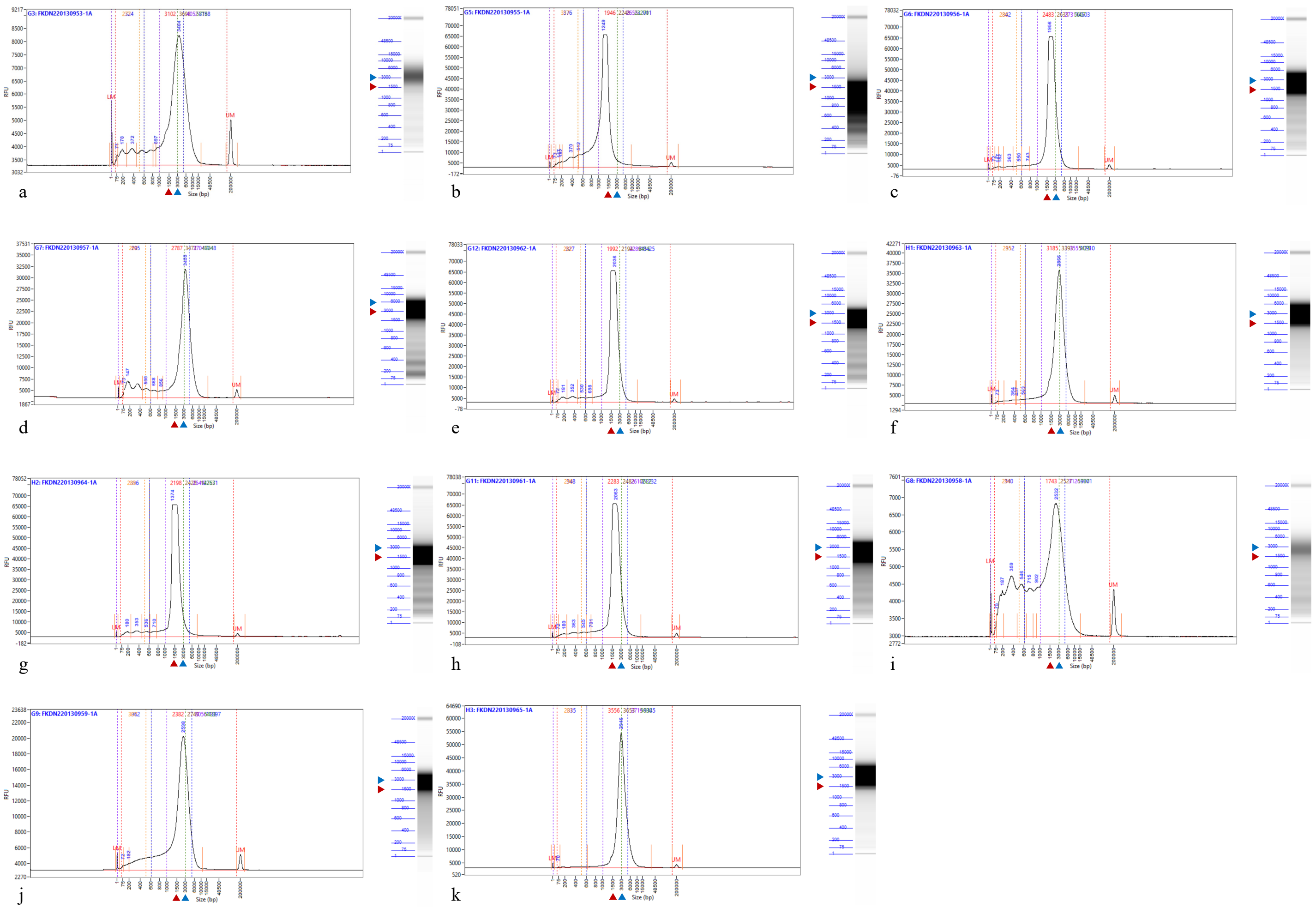

Figure S1. The fragment size distribution of the shed skin DNA extracted by the standard method. (a) PAGU0069, (b) PAGU0091, (c) PAGU0092, (d) PAGU0093, (e) PAGU0106, (f) PAGU0114, (g) PAGU0118, (h) PAGU0120, (i) PAGU0126, (j) PAGU0129, (k) PAGU0133.Picture comes from DNA sample quality inspection report done by Novogene. The x-axis (bp): fragment size distributed in the capillary sample. The y-axis (RFU): RFU value refers to the real-time fluorescence signal intensity of the sample in the process of capillary separation. The higher the RFU value, the higher the sample concentration. The right side shows the simulated gel map and the corresponding Ladder size value. The red triangle: 1,500 bp. The blue triangle: 3,000 bp.

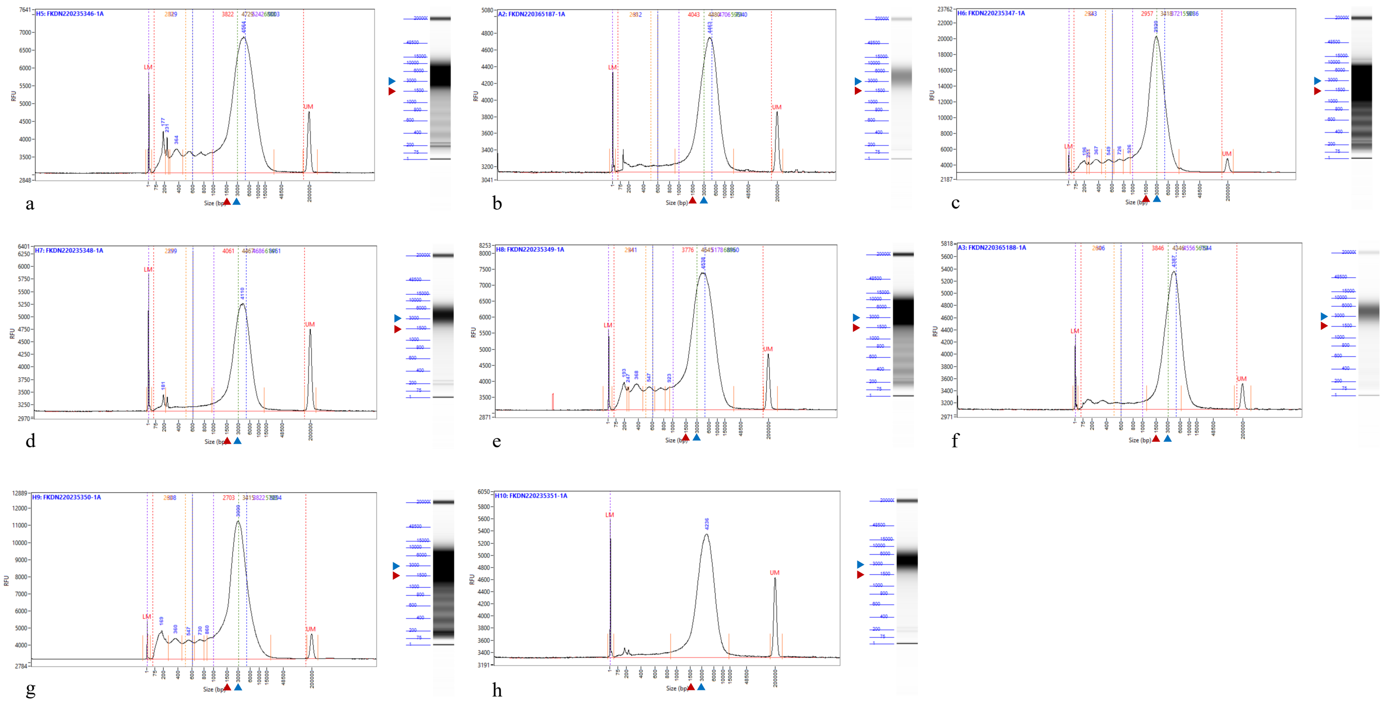

Figure S2. The fragment size distribution of the shed skin DNA extracted by the modified method. (a) PAGU0092-30min, (b) PAGU0093-30min, (c) PAGU0126-30min, (d) PAGU0129-30min, (e) PAGU0092-60min, (f) PAGU0093-60min, (g) PAGU0126-60min, (h) PAGU0129-60min. Picture comes from DNA sample quality inspection report done by Novogene. The x-axis (bp): fragment size distributed in the capillary sample. The y-axis (RFU): RFU value refers to the real-time fluorescence signal intensity of the sample in the process of capillary separation. The higher the RFU value, the higher the sample concentration. The right side shows the simulated gel map and the corresponding Ladder size value. The red triangle: 1,500 bp. The blue triangle: 3,000 bp.
